# Mesenchymal Stem Cells in Preclinical Infertility Cytotherapy: A Retrospective Review

**DOI:** 10.1155/2021/8882368

**Published:** 2021-05-17

**Authors:** Zhuo Chang, Hui Zhu, Xueming Zhou, Yang Zhang, Bei Jiang, Shuoxi Li, Lu Chen, Xue Pan, Xiao-ling Feng

**Affiliations:** ^1^Heilongjiang University of Chinese Medicine, Harbin, Heilongjiang, China; ^2^First Affiliated Hospital, Heilongjiang University of Chinese Medicine, Harbin, Heilongjiang, China; ^3^Jiamusi College of Heilongjiang University of Traditional Chinese Medicine, Jiamusi, Heilongjiang, China; ^4^Beijing University of Traditional Chinese Medicine, Beijing, China

## Abstract

Infertility is a global reproductive disorder which is caused by a variety of complex diseases. Infertility affects the individual, family, and community through physical, psychological, social and economic consequences. The results from recent preclinical studies regarding stem cell-based therapies are promising. Stem cell-based therapies cast a new hope for infertility treatment as a replacement or regeneration strategy. The main features and application prospects of mesenchymal stem cells in the future of infertility should be understood by clinicians. Mesenchymal stem cells (MSCs) are multipotent stem cells with abundant source, active proliferation, and multidirectional differentiation potential. MSCs play a role through cell homing, secretion of active factors, and participation in immune regulation. Another advantage is that, compared with embryonic stem cells, there are fewer ethical factors involved in the application of MSCs. However, a number of questions remain to be answered prior to safe and effective clinical application. In this review, we summarized the recent status of MSCs in the application of the diseases related to or may cause to infertility and suggest a possible direction for future cytotherapy to infertility.

## 1. Introduction

Infertility is a common and complex disease characterized by the failure to establish a clinical pregnancy after 12 months of regular and unprotected sexual intercourse [1]. According to the latest statistics, infertility is estimated to affect 9% to 18% of reproductive-aged couples worldwide, and the incidence is increasing each year [2]. There is a growing body of data to support the idea that infertility has implications that go beyond patients' immediate reproductive needs. Infertility is a medical and social condition that impacts millions of women worldwide. As a consequence, patients feel substantial economic, mental, and social pressure during the treatment, management, and research of infertility, leading to stress and decreased quality of life. Infertility has negative consequences for demographics, socioeconomics, and health. As a global medical problem, infertility has drawn much attention and research in the medical field. Because of their biological characteristics, mesenchymal stem cells can regulate immunologic mechanisms and repair the uterus, ovaries, and oviductal tissue. MSCs can also play a positive role in the treatment of azoospermia and oligozoospermia. Therefore, in this article, we summarize the current status of mesenchymal stem cell therapies in infertility pathophysiology and discuss potential areas of further research in regenerative medicine. This review culminates with the current limitations of our understanding, which may be the impetus for future studies.

### 1.1. The Etiology of Infertility

At present, the causes of infertility have been discovered: infertility does not always originate in females. Approximately one-third of infertility cases involve females. In the remaining two-thirds of cases, the reason involves both males and females, or no cause can be found [3, 4]. Infertility is classified as primary and secondary infertility, each of which has different origins. The roots of primary infertility are always associated with genetic abnormalities [5]. Secondary infertility may be treated by the potential therapeutic effect of MSCs and is closely related to a variety of acquired diseases such as abnormal sperm production or function, problems with the delivery of sperm, overexposure to certain environmental factors, or damage related to certain drugs, xx. in men [6]. Moreover, the reasons for female infertility include ovulation inhibition, uterine fibroids, injury, or obstruction of the fallopian tubes, endometriosis, or damage related to specific drugs, xx. [7–9]. However, approximately 5% to 10% of infertile women may have underlying genetic abnormalities such as chromosome aberrations, single or multiple gene mutations, and polymorphisms [10]. Further, underlying environmental, age, and psychological factors may also play roles in infertility [11]. Recent evidence demonstrates that epigenetic modifications also have ramifications for infertility [12]. The above elements may affect the formation of oosperms or the process of embryo implantation, leading to the occurrence of infertility.

### 1.2. Treating Infertility

The goal of fertility treatment is to become pregnant and achieve a successful outcome. Currently, considerable progress has been made in the treatment of infertility. Women with anovulation may be treated with clomiphene to induce ovulation [13]. Treatment of tubal obstruction generally requires referral for subspecialty care and hydrotubation [14]. Unexplained infertility in women or men may be managed with another year of unprotected intercourse or may proceed to assist reproductive technologies, such as intrauterine insemination or in vitro fertilization, ovum or sperm donation, pregnancy surrogacy, and preimplantation treatment or diagnosis [15, 16]. However, the success of these therapeutic approaches is limited due to related complications, a long process, and ethical issues. Thus, there is an unmet medical need to develop more effective therapeutic approaches for infertility.

Recent progress in studies has shown the potential of stem cells for therapeutic purposes, and recent studies in the field of mesenchymal stem cell biology have provided new perspectives and opportunities for the treatment of infertility disorders. An increasing number of scholars are concerned about the role of mesenchymal stem cells in the treatment of infertility. Because of their biological characteristics, mesenchymal stem cells can regulate immunologic mechanisms and repair the uterus, ovaries, and oviductal tissue. MSCs can also play a positive role in the treatment of azoospermia and oligozoospermia. Therefore, in this article, we summarize the current status of mesenchymal stem cell therapies in infertility pathophysiology and discuss potential areas for further research needed in regenerative medicine. This review culminates with the current limitations to our understanding, which may be the impetus for future studies.

### 1.3. Defining and Identifying MSCs

Mesenchymal stromal cells (MSCs) were initially discovered by Prockop in the mid-1970s as a small fraction of heterogeneous cells from bone marrow, which is readily isolated [17]. Mesenchymal stem cells (MSCs) are somatic stem cells that have the capacity for self-renewal, multidirectional differentiation, and immunoregulation [18, 19]. MSCs are also referred to as multipotent mesenchymal stromal cells, which hold great potential for application in tissue engineering and regenerative medicine and have been the focus of recent research. MSCs are considered “seed cells” because they have the ability to differentiate into all cell types within one particular lineage and can become any type of cell according to the location where they have been transplanted in [20]. MSCs can not only repair, renew, and replicate themselves but are also induced to differentiate into a variety of cells in vitro, such as osteocytes, adipocytes, and chondrocytes [21]. Furthermore, MSCs also play an immunoregulatory role in T cells and B cells. MSCs can be isolated from multiple tissues including the following: skeletal muscle, adipose tissue (AT), marrow, periodontal ligaments, cervical tissue, menstrual blood, umbilical cord (UC), umbilical cord blood (UCB), amniotic fluid, and the placenta (PL), as well as fetal tissues such as blood, liver, and BM [22–26]. Because of these limitations, MSCs are more readily available and easier to accept in ethics than embryonic stem cells.

To date, there is no definite marker of MSCs and no gold standard for identifying them. In 2006, the International Society for Cellular Therapy established three minimum criteria to fulfill certain characteristics to identify MSCs [27]. First, under the condition of standard culture in vitro, MSCs must have adherent properties to plastics [28]. Second, MSCs must be positive for CD105, CD73, and CD90, with an expression of more than 95%; the cells should also be negative for CD45, CD34 or CD11b, CD79a or CD19, and HLA-DR, and the negative expression rates should be more than 98% [29]. Third, these cells must be capable of differentiating into osteoblasts, adipocytes, and chondroblasts in vitro [30]. Recently, some studies have found that Nestin is positive in menstrual blood-derived mesenchymal stem cells, and the positive expression of Nestin suggests that these cells have the potential to transform into nerve cells. This finding also implies that Nestin may become a new marker of MSCs [31]. However, there are currently no specific markers of MSCs, so it is difficult to locate and track MSCs in vivo.

### 1.4. Characteristics of MSCs

In most cases, MSCs cultivated in vitro possess four biological properties that qualify them for use in cellular therapy [32–35]: (a) broad potential of differentiation, (b) secretion of trophic factors that favor tissue remodeling, (c) immunoregulatory properties, and (d) low immunogenicity. These characteristics bring a unique advantage to MSCs in the field of cellular therapies and make MSC potential tools in many conditions. The low immunogenicity of MSCs provides advantageous conditions for allogeneic cell transplantation and reduces the chance of immune rejection after cell transplantation [36, 37]. Because of the differentiation potential of MSCs, the cells can differentiate into different mesodermal cells (e.g., adipocytes, chondrocytes, osteocytes, fibroblasts, and myocytes) [19]. Therefore, MSCs were initially used to treat osteogenesis imperfecta [38] and myocardial injury [39]. MSCs can secrete cytokines and growth factors with paracrine effects that favor the regeneration of damaged tissues [17, 34]. Additionally, their benefits arise primarily from the secretion of trophic factors and immunoregulatory capacity. In recent years, some studies have extensively investigated the immunosuppressive potential of MSCs in vitro and in vivo [40]. Although the underlying mechanisms of immunomodulation have not yet been elucidated [41], it is likely to occur by the secretion of some soluble factors and cell contact-dependent mechanisms in response to other cells. These biological characteristics enable MSCs to become a potential tool in infertility treatment and play a positive role.

### 1.5. Mechanisms of MSCs in the Treatment of Infertility

The reason why MSCs can have great potential in the treatment of infertility is related to their biological characteristics.

#### 1.5.1. Differentiative Capacity

MSCs should have the ability to differentiate into various cell types such as epithelial, stromal, and endothelial cells [42]. This differentiation potential allows MSCs to repair tissue damage that leads to infertility. In 2018, Zheng et al. were the first to demonstrate the ability of MSCs to differentiate into endometrial cells in the laboratory [43]. However, although some studies have reported that MSCs improve ovarian function and help ovarian functional recovery, previous studies have also found that the number of differentiated MSCs is not sufficient to account for observed improvements in fertility, and controversy remains regarding the differentiation of MSCs into oocytes after migrating to target tissue [44].

#### 1.5.2. Secretory Capacity

Many studies have shown that the reproductive treatment effect of MSCs is linked to their secretome, which is rich in bioactive factors, including insulin-like growth factor (IGF), vascular endothelial growth factor (VEGF), and other growth factors that restore tissue function [45]. These remarkable effects of BMSCs may be due to angiogenic and growth factors secreted from these cells [46]. The paracrine activity of MSCs has a more significant effect on ovary function than its stimulatory effects on cell growth and differentiation [47]. This potential mechanism of MSC activity can regulate immune responses and inflammation, induce regeneration and angiogenesis, as well as inflammation, improve injured tissues, and stimulate progenitor cells to differentiate into specific tissue cells [48]. These pathways also enhance ovarian function. For example, MSC treatment can restore ovarian structure by inducing VEGF expression [49].

Third, miRNA and exosome transfer may be a novel mechanism by which MSCs exert functions. It appears that a proinflammatory environment can boost MSC mitochondrial transfer into T cells, in turn training immune cells to control the inflammatory response [50]. One study recently revealed the inflammation-driven mitochondrial transfer of MSCs to reproductive system cells, including oocytes [51]. Additionally, some studies have detected the role of miRNAs carried by MSCs in promoting the recovery of ovarian function in a POF animal model [52, 53]. A more recent study described that BMSC-derived exosomal miR-144-5p can restore ovarian function by regulating PTEN after chemotherapy-induced POF in rats [53]. These findings indicate that miRNA-regulated gene expression underlies MSC-based therapy outcomes and confirm that miRNAs play a critical role in regulating stem cell differentiation and regeneration by inhibiting target mRNA translation [54].

### 1.6. The Endometrium and MSCs

The uterus is a place for embryos to implant, grow, and develop. Composed of a compact layer, a spongy layer, and a basal layer, the endometrium is a layer of tissue that forms the inner wall of the uterus. The spongy layer and compact layer are collectively called functional layers, which come off periodically with changes in the level of hormone [55–58], while the basal layer remains. Because the endometrium is the basis for establishing and maintaining pregnancy, and defects in the endometrium will cause a variety of diseases, a typical case is infertility. Defects of the endometrium that contribute to infertility have been linked to a wide array of disorders, including endometriosis, endometrial injury, endometrial fibrosis, and endometrial adhesions, all of which can reduce endometrial receptivity and negatively impact implantation [59–61]. Compromised receptivity of the endometrium is a major cause of unexplained infertility, implantation failure, and subclinical pregnancy loss [62–64]. As the diseases linked to endometrial defects easily recur and have many sequelae, consequently, searching for a safe and effective treatment which can repair the defect is being necessary in the reproductive field [65]. At present, mesenchymal stem cell therapy has achieved good results in the field of repairing endometrial defects, especially for injury and endometrial adhesion. However, but the role of endometriosis is still controversial.

The role of MSCs in repairing endometrial defects, injuries, and adhesions (among other aspects) mainly benefits from proliferation and differentiation, as well as the paracrine secretion of multiple factors that stimulate angiogenesis, reduce fibrosis, and promote endogenous cell proliferation to affect tissue repair [66]. MSCs can express markers of human endometrial stromal cells and can transform into them under certain conditions in vitro [67, 68]. Therefore, the transplantation of MSCs can also increase the thickness and receptivity of the endometrium. Zhang et al. found that MSCs have the potential to differentiate into endometrial epithelial cells under estrogen induction conditions in vitro [69]. Yang et al. [70] also showed that the effect of mesenchymal stem cells, isolated from Wharton jelly of the umbilical cord (WJ-MSCs), on ameliorating damaged human endometrial stromal cells (ESCs), is obvious. WJ-MSCs can repair injured endometrial stromal cells induced by mifepristone. When damaged ESCs were cocultured with WJ-MSCs, the proliferation of these damaged cells significantly increased, and the apoptosis percentage decreased. Some scholars suggest that the repair mechanism of MSCs in vivo is due to factors such as fibroblast growth factor, insulin-like growth factor- (IGF-) 1, TGF-*β*1, and VEGF (among others). These factors can promote the proliferation and migration of local endometrial cells, repair endometrial injury, and recuperate one's ability to conceive [71]. Nesrine et al. and Wang et al. concluded that the transplantation of MSCs can provide a highly effective, alternative regenerative agent in intrauterine adhesion (IUA) and endometrial adhesion treatment [72, 73]. MSC therapy has been applied to treat Asherman's syndrome, which is linked to infertility. Further, MSC transplantation therapy can reduce the degree of inflammation after adhesiolysis and promote endometrial regeneration [74]. In addition, a study showed that autologous in utero MSC transplantation facilitated IVF in the case of a woman with severe endometrial adhesion following dilation and curettage [75].

Endometriosis is an inflammatory disease marked by the ectopic growth of endometrial cells, which can lead to chronic pelvic pain and infertility. It affects ~10% of reproductive-age women, but might account for a greater number of cases of infertility, as studies have suggested that 25% to 50% of infertile women will have endometriosis, and that 30% to 50% of women with endometriosis will be infertile [76–79]. Currently, the cause of endometriosis-induced infertility remains elusive, but it has been confirmed that the causes include adhesions, the malfunction of immunity, and altered endometrial receptivity. According to reports, it impacts 5% to 10% of women of reproductive age and 20% to 50% of women with infertility [80]. Palindromia and therapy resistance are very common, although some medical and surgical treatments are used to reduce inflammation and remove ectopic lesions [81]. Some studies have revealed that ovulatory suppressive medications such as oral contraceptive pills, GnRH agonists, and danazol do not improve spontaneous pregnancy and fertility rates for infertile women with endometriosis seeking conception. Surgical treatment and assisted reproductive technology (ART) also have some limitations [82]. Therefore, there is an urgent need for new therapies for endometriosis. The transplantation of MSCs is also considered as a potential cell therapy, but unlike its relevant role in the treatment of endometrial defects, the application of MSCs for treating endometriosis, particularly endometriosis-associated infertility, has caused controversy. The reason for this controversy is that stem cells are involved in the pathogenesis of the disease. For example, several reports have indicated that bone marrow-derived MSCs can differentiate into carcinoma-associated fibroblasts and promote tumor growth [83, 84]. Additionally, MSCs from adipose tissue have been demonstrated to promote cancer proliferation and metastasis [85, 86]. Because endometriosis behaves similarly to cancer in many ways, considering the risk of disease, it is vital to determine the optimal cell therapy type. Further, Abomaray et al. found that MSCs from allogeneic adipose tissue (Ad-MSCs) significantly increased the proliferation, adhesion, and survival of ESCs and also decreased apoptosis. The data imply that allogeneic Ad-MSCs should not be considered as a potential therapy for endometriosis, since they may support the pathology by maintaining and increasing the growth of ectopic endometrial tissue [87]. Moreover, several findings suggest that BMSC replacement is capable of improving endometrial dysfunction and infertility in patients with Asherman's syndrome by increasing endometrial receptivity, inducing angiogenesis, and suppressing fibrosis [88–90].

### 1.7. MSC and Ovarian Function

Generic ovulatory dysfunction is one of the important causes of infertility, and abnormal ovarian function can result in infertility as well as pregnancy loss and miscarriage. The main diseases of ovarian dysfunction related to infertility include premature ovarian failure (POF) and polycystic ovary syndrome (PCOS).

Both hypogonadotropism and hypergonadotropism concur with ovary failure in approximately 20% of infertile women [91]. Among them, POF accounts for approximately 1%. POF refers to amenorrhea caused by ovarian follicular degeneration or dysfunction before the age of 40. In most cases, decreased ovarian function and premature ovarian failure are associated with chemotherapy, autoimmune diseases, and operation of the ovary, among other conditions [92]. Decreased ovarian function not only leads to female infertility, but also affects long-term fertility ability and pregnancy outcomes [93]. Currently, the clomiphene citrate (CC) has been widely adopted in the past for generic ovulatory dysfunctions. Modern treatments of infertility caused by ovulation disorders include ovulation induction, in vitro oocyte fertilization, egg donation, and pregnancy surrogation [94, 95]. Although the above methods are helpful in restoring the fertility of patients with decreased ovarian function, they cannot fundamentally restore ovarian function, and these treatments may cause adverse reactions and complications. With the in-depth study of MSCs, scholars found that MSCs may become a new potential tool to treat ovarian dysfunction and restore fertility. It has been previously reported that the transplantation of MSCs from multiple sources can repair damaged ovarian tissues and survival, and MSC transplantation can also improve ovarian function in premature ovarian insufficiency and aging animal models by increasing folliculogenesis, decreasing granulosa cell apoptosis and the extent of ovarian fibrosis, and modulating cytokine expression [96–98]. This mechanism may be related to its own differentiation and secretion capacity and the function of regulating immunity [99, 100]. Zhang et al. [101] screened the differentially expressed proteins in in frozen-thawed ovarian tissues cotransplanted with MSCs by cytokine microarrays and established that the ability of MSCs to release paracrine factors with proangiogenic functions may be the molecular mechanism by which MSCs promote angiogenesis and follicle survival in transplanted ovarian tissues. In animal models, significant results have been achieved in improving ovarian function and restoring fertility by transplanting MSCs. Zhu et al. [102] transplanted human umbilical cord mesenchymal stem cells (hUC-MSCs) into premature ovarian failure rats induced by injecting cyclophosphamide and evaluated whether the levels of sex hormones in rats were significantly increased, and some rats even recovered fertility. Wang et al. [103] demonstrated that blood-derived mesenchymal stem cell transplantation can repair the ovarian function of a POF animal model. Wang et al. [104] studied and discussed the effect and mechanism of MSCs in POF animal models and proved that MSCs had a good therapeutic effect on infertility caused by POF or ovarian injury and could improve ovarian function. Yuji et al. [105] discovered that MSCs were capable of inducing angiogenesis and restoring the number of ovarian follicles and corpus lutea in ovaries; as for tumor formation, death or any obvious side effects were not found in animal models that underwent transplantation. Further, Kozub et al. [106] compared the ability to recover ovarian function in mice transplanted with MSCs from different sources and revealed that MSCs isolated from placenta had the strongest recovery ability. In May 2017, Wu et al. [107] demonstrated that postmenopausal female mice were able to produce healthy offspring after allogeneic oogonial stem cell transplantation; no genetic defects were observed. Collectively, the results of these preclinical studies provide a certain basis for the safety and ability to promote the restoration of fertility in patients with premature ovarian failure of stem cell therapy. According to recent findings, human MSCs significantly restore hormone secretion, survival rate, and reproductive function in POF mice. Notably, the transplanted mice generated new offspring. [108]. This outcome will serve as an important foundation for MSCs in stem cell therapy in infertility cytotherapy. Although stem cell therapy has not been widely promoted in clinical practice and a major number of clinical trials have not yet acquired full regulatory approval for validation as stem cell therapies, there is still encouraging news. According to a report from 2016 [109], a woman with POF successfully gave birth to a baby after transplanting autologous bone marrow-derived mesenchymal cells into the ovary. This concept was discussed as a possible ray of hope for women with POF [110].

PCOS is a common endocrine metabolic disorder affecting 5% to 15% of women and accounts for >27% of infertility cases [111, 112]. According to the Rotterdam code, PCOS should be considered present when two of three criteria have been met: ovulatory dysfunction, hyperandrogenism, and/or polycystic-appearing ovarian morphology on pelvic ultrasonography [113]. Women with PCOS, even after ovulation has been restored, will have an increased risk of spontaneous miscarriage, with lower cumulative pregnancy rates compared to other subfertile populations [114, 115]. To date, there is an unmet medical need to develop more effective therapeutic approaches for PCOS. Some studies suggest that MSCs may play a potential role in the treatment of PCOS. The findings point to the work of Xie et al. [116], who examined a potential role for MSCs that could improve the pathological changes of PCOS mice induced by dehydroepiandrosterone (DHEA), including ovarian histopathology and function. Moreover, this study showed that the administration of MSCs significantly downregulated the expression of proinflammatory factors and fibrosis-related genes in ovarian and uterine tissues and affected the systemic inflammatory response. These results suggest that MSC treatment could alleviate ovarian dysfunction by inhibiting ovarian local and systemic inflammatory responses. Moreover, compared with the PCOS rat model without intervention methods, the female PCOS rat model injected with menstrual blood-derived MSCs expressed lower follicle-stimulating hormone (FSH) and higher serum luteinizing hormone (LH). The therapeutic method of MSC transplantation can also reduce the number of follicles in PCOS rats [117].

### 1.8. MSC and Fallopian Tubes

The fallopian tube is a passive channel for gametes and early embryos. Moreover, the fallopian tube plays a key role in many reproductive functions such as sperm transport and capacitation, ova retrieval and transport, fertilization, and embryo nourishment and transport. Fallopian tube factor is also a common cause of infertility, mainly due to basic salpingitis and tubal injury [118]. Fallopian tube inflammation and injury will not only weaken cilia swing and reduce the ability to transport pregnant eggs but also eventually lead to tubal blockage due to hyperplasia and prevent conception [119]. Therefore, one important way of improving the success rate of women's pregnancy is to choose safe, effective, and uncomplicated quality methods to treat tubal injury and inflammation as soon as possible.

Mounting evidence from studies in mice over the past decade supports the idea that mesenchymal stem cells (MSCs) have a strong tendency to improve tissue destruction in response to injury and disease; their therapeutic potency has been demonstrated in animal models of ovarian failure [120], myocardial infarction [121], lung injury [122], and a thin endometrium [123]. In recent years, the application of MSCS in the treatment of fallopian tube diseases has received increasing attention. According to findings obtained from animal experiments, MSC transplantation can improve tubal injury and may provide a new treatment idea for infertility caused by tubal factors [124]. Abd-Allah et al. [125] treated a mouse model of chronic salpingitis caused by a chlamydia infection by injecting human umbilical cord MSCs through the vagina; the outcomes indicated that the degrees of salpingitis and adhesion in mice after transplantation were reduced. This may be because some cytokines secreted by MSCs inhibit the inflammatory process or repair fallopian tube injury by their own differentiation ability. Almasry et al. [126] observed that after transplanting human bone marrow mesenchymal stem cells into fallopian tube injured rats, the injured tissues were repaired, and the transplanted MSCs were homed to the distal end of the fallopian tube. This study strongly supports the notion that MSCs could exhibit their restorative effect on fallopian tubes through their ability to activate resident stem cells in the distal tubal end, alternatively, mediating the expression of VEGF and PCNA and influencing tissue apoptosis. In parallel, human umbilical cord mesenchymal stem cells (hUC-MSCs) have been confirmed to play a positive role in restoring the reproductive ability of SD female rats with chronic salpingitis. Moreover, hUC-MSC therapy is highly safe [127]. However, there are still some studies on the efficacy of MSC treatment, such as Luo et al. [128], who treated acute chronic fallopian tube rat models induced by a mixed bacterial fluid through intraperitoneal injection of human umbilical cord MSCs. The study revealed that the acute group could restore the function of the fallopian tube and improve fertility function, but the chronic group had a poor therapeutic effect.

In summary and considering the experimental methods, the authors believe MSCs can repair injured fallopian tubes through their own differentiation and regeneration ability and play a certain role in the treatment of fallopian tube inflammation after healing and chronic fallopian tube inflammation through its immunomodulatory or secreted anti-inflammatory factors. Notwithstanding, in the treatment of acute fallopian tube inflammation, due to the inconvenience in obtaining materials and a lack of pertinence, it has drawbacks compared with the currently commonly used antibiotic treatment scheme.

### 1.9. MSC and Male Infertility Factors

Male infertility is one of the most important issues that affect the personal and social life of a family and its members. Given the significant contribution of male factors to infertility in couples, a lack of understanding of the underlying mechanisms seems to be a critical challenge. Numerous causes are responsible for male infertility, such as infections, smoking, heavy metal exposure, radiation, and certain drugs, in addition to anatomical defects and endocrinopathies [129]. Current studies have shown that some common medical treatments, including surgery [130], and hormone and drug therapies, have achieved certain results in improving the quantity and quality of spermatozoa. However, the therapeutic effect has limitations and cannot be fundamentally treated, so new treatments still need to be sought seek a new treatment. In recent years, after identifying the paracrine function and the high capability of mesenchymal stem cells, a number of scientists have proposed the use of stem cells and cell-based therapies as a possible new therapeutic choice for male infertility [131]. Stem cells have been adopted relatively recently in andrology studies on infertility as potential therapeutic agents. Notwithstanding, it is still in the animal experiment stage. That said, with a deep understanding of its great potential and achievements, it is hoped that novel and effective methods for the treatment of male infertility based on MSCs will emerge in the near future.

Some scholars supported the idea that stem cells have huge potential prospects for the treatment of erectile dysfunction (effective dysfunction, ED). In addition, stem cells may also be used to treat late onset hypogonadism (LOH), a disease associated with low serum testosterone levels [131]. Numerous studies have found that the mechanism of restoring male fertility through MSCs transplantation may be related to the ability of MSCs to differentiate into spermatogonia. Many studies have confirmed that MSCs can differentiate into GCs (germ cells) in vitro [132–136]. Further, some studies have some reported that transplanted MSCs could fully differentiate into sperm cells and regenerate spermatogenesis [137, 138]. These conditions have become the basis for MSCs to restore male fertility. A study showed that BM-MSCs can promote spermatogenesis after transplantation into the testes of busulfan-induced infertile male hamsters [139]. Zhang et al. [140] found that rat autologous BM-MSCs could survive in the testes and differentiate into spermatogonia after transplantation into the testis of infertile male rats. From this result, scholars surmised that optimization of the MSC culture conditions prior to transplantation may eventually be achieved and solve the problem of infertility. Tamadon et al. confirmed that MSCs derived from adipose tissue also have potential use in the treatment of azoospermia using rat disease models [141]. Although MSC transplantation has been proven to restore male fertility, it is still necessary to study and observe whether fertility-restored rats can successfully mate, produce offspring, and whether their offspring are healthy [142, 143].

### 1.10. Application of Mesenchymal Stem Cells in Clinical Therapy

Although the treatment of infertility by MSCs is mainly focused on preclinical study at present, there are still a few experiments that have applied this treatment method to clinical practice and have achieved good efficacy. Previous studies have found that MSC therapy can significantly improve infertility caused by intrauterine adhesion, and the total natural pregnancy rate after treatment can reach 27.78%. The effective rates of bone marrow-derived mesenchymal stem cells (BMD-MSCs), menstrual blood-derived mesenchymal stem cells (Men-SCs), umbilical cord-derived mesenchymal stem cells (UC-MSCs), and adipose-derived mesenchymal stem cells (ADSCs) were 18.75% [144], 14.29% [145], 38.46% [146], and 20% [147], respectively. However, the clinical research on MSC therapy of male infertility is still in its infancy. Up to now, there are 6 studies on MSC treatment of male infertility on the website of Clinical Trials in the United States, but no clear statistical clinical effective rate has been announced [148]. Although there are few studies on the clinical application of MSCs, it can still be seen that they have great potential prospects in the treatment of infertility diseases.

## 2. Conclusion

Infertility is both a medical and a social problem that affects a large male and female population worldwide. It is associated with a number of pathophysiological conditions, and its pathogenesis is frequently undefined, with relative uncertainty in establishing appropriate treatment choices. In recent years, with the development of research on stem cells, increasing evidence has shown that stem cells, especially mesenchymal stem cells, may become a potential tool for the treatment of infertility. Stem cell-based therapy, as a modality of regenerative medicine, is considered one of the most promising disciplines in modern science and medicine. Such advanced technology offers endless possibilities for transformative, potentially curative treatments for difficult diseases. This article summarizes the current research progress on mesenchymal stem cells in diseases related to infertility; advancements indicate that this emerging evidence contributes to solving problems related to infertility. Furthermore, these data indicate the potential to harness the properties of stem cells for clinical applications and are also reviewed. Although remarkable achievements have been made, more research support and improvement are needed.

## Data Availability

The data supporting this systematic review are from previously reported studies and datasets, which have been cited. Previously reported data were used to support this study and are available at DOI. These prior studies and datasets are cited at relevant places within the text as references [#-#].
